# Serum Ceruloplasmin Levels Correlate Negatively with Liver Fibrosis in Males with Chronic Hepatitis B: A New Noninvasive Model for Predicting Liver Fibrosis in HBV-Related Liver Disease

**DOI:** 10.1371/journal.pone.0077942

**Published:** 2013-10-25

**Authors:** Da-Wu Zeng, Yu-Rui Liu, Jie-Min Zhang, Yue-Yong Zhu, Su Lin, Jia You, You-Bing Li, Jing Chen, Qi Zheng, Jia-Ji Jiang, Jing Dong

**Affiliations:** 1 Liver Center, First Affiliated Hospital, Fujian Medical University, Taijiang District, Fuzhou, Fujian Province, China; 2 Department of Pharmacy, First Affiliated Hospital, Fujian Medical University, Taijiang District, Fuzhou, Fujian Province, China; Drexel University College of Medicine, United States of America

## Abstract

**Aims:**

This study aimed to investigate associations between ceruloplasmin (CP) levels, inflammation grade and fibrosis stages in patients with chronic hepatitis B (CHB) and to establish a noninvasive model to predict cirrhosis.

**Methods:**

Liver biopsy samples and sera were collected from 198 CHB patients randomized into a training group (n=109) and a validation group (n=89). CP levels were determined using nephelometric immunoassays. Relationships between CP and liver inflammation and fibrosis were analyzed by Spearman rank correlation. Receiver operator characteristic (ROC) curves were used to evaluate the diagnostic value of CP for determining liver fibrosis in CHB. The liver pathology-predictive model was built using multivariate logistic regression analysis to identify relevant indicators.

**Results:**

CP levels were lower in males than in females, lower in patients with inflammation stage G4 compared to other stages and lower in cirrhotic compared to non-cirrhotic patients. Using area under the curve (AUC) values, CP levels distinguished different stages of inflammation and fibrosis. Multivariate analysis showed that CP levels were all significantly associated with cirrhosis in males. A model was developed combining routine laboratory markers APPCI (alpha-fetoprotein [AFP], prothrombin time, and platelets [PLT] with CP) to predict fibrosis in CHB patients. The APPCI had a significantly greater AUC than FIB-4 (aspartate aminotransferase [AST]/ alanine aminotransferase [ALT]/PLT/age), APRI (AST/PLT ratio index), GPI (globin/PLT), and APGA (AST/PLT/gammaglutamyl transpeptidase [GGT]) models (all *P*-values<0.001).

**Conclusions:**

CP levels correlate negatively and indirectly with inflammation and fibrosis stages in male CHB patients. The APPCI model uses routine laboratory variables with CP to accurately predict liver fibrosis in CHB.

## Introduction

The World Health Organization estimates that the hepatitis B virus (HBV) causes chronic infection in 350-400 million people worldwide, of whom 75% are Asian [1,2]. Up to 40% of patients with chronic hepatitis B (CHB) progress to chronic end-stage liver disease or hepatocellular carcinoma (HCC) during their lifetime [3]. Liver histology of CHB patients with repeated hepatitis flares shows increased necroinflammation, which may lead to increased fibrogenesis and disease progression [4]. However, some patients with persistently normal levels of serum alanine transaminase (ALT) may also progress to advanced fibrosis or cirrhosis [5-7]. 

Liver fibrosis is part of the natural wound healing response when chronic HBV infection has caused parenchymal injury, and this pathogenic process may eventually result in cirrhosis [4,8]. The histologic grade of inflammation and liver fibrosis stage can influence the clinical management in these patients, suggesting that determining the extent of liver fibrosis is critical for the prognosis and clinical management of CHB [4,9]. Based on the European Association for The Study of the Liver (EASL) Clinical Practice Guidelines (CPG)[10], treatment for CHB is recommended for: 1) patients with elevated ALT when there is clinical evidence of moderate to severe active necroinflammation and/or at least moderate fibrosis on liver biopsy; and 2) patients with normal ALT levels in the presence of compensated cirrhosis and detectable levels of HBV DNA. There is currently no single routine laboratory parameter that can be reliably used to reflect fibrosis. Liver biopsy remains the gold standard in assessing fibrosis and cirrhosis, however, up to 2% of patients develop complications from this procedure [11]. The cost, invasiveness and risks associated with liver biopsy preclude its use for monitoring patients’ response to antiviral therapy. Therefore, the recent focus has been on developing new predictive models of fibrosis, including several non-invasive models for evaluating fibrosis specifically in HBV patients [12-14]. However, most studies of model development have focused on noninvasive markers for evaluating patients with chronic hepatitis C, including FIB-4 (AST/ ALT/ PLT/age) [15], FibroTest (α-2-macroglobulin, γ-glutamyl transpeptidase [GGT], apolipoprotein, haptoglobin, total bilirubin, age and gender) [16], APRI (AST/PLT ratio) [17], European Liver Fibrosis (ELF) score (hyaluronate [HA], procollagen III amino terminal peptide, and tissue inhibitor of metalloproteinase 1 [TIMP-1], age) [18]; and Hepascore (bilirubin, GGT, hyaluronate, α-2-macrogluobulin, age and gender) [19]. In a comprehensive review of noninvasive methods for assessing liver disease in HBV and HCV patients, Castera [20] noted that the accuracy and applicability of serum biomarker assays vary between HBV and HCV patients, and some combined noninvasive models that have shown increased diagnostic accuracy for HCV have not been validated in HBV patients. Fibrometer, which combines platelet count, prothrombin index, AST, α-2-macroglobulin, hyaluronate, urea and age, has been evaluated in both HBV and HCV patients [21]. The cost of certain methods, especially when performance requires specific equipment or non-routine laboratory tests, and the ease of measurement of predictive biomarkers are other factors that have been suggested to limit the use of some non-invasive models in some institutions [12,21-23], even though all are less expensive than liver biopsy.

Ceruloplasmin (CP) is a copper-containing glycoprotein synthesized predominantly in the liver. It is the major carrier for copper in the blood, accounting for 90% of the circulating copper in normal individuals [24]. Copper homeostasis is disrupted in a number of liver diseases [25,26]. Serum CP is typically decreased in patients with Wilson’s Disease (WD) [27], and other conditions characterized by marked renal or enteric protein loss, including during severe end-stage liver disease of any etiology and in some rare neurologic diseases [28]. Interestingly, patients with severe hepatitis were shown to have significantly lower levels of CP compared to patients with other liver diseases, except for WD [29]. However, the relationship between CP and liver inflammation and fibrosis stages remains unclear and it is imperative to explore whether low levels of serum CP indicate ongoing liver damage and repair in chronic liver diseases such as CHB. 

Although CP levels were previously shown to be decreased in patients with severe hepatitis [29], no studies to date have evaluated CP levels as a biomarker to predict fibrosis. In the present study, we aimed to analyze serum CP levels in CHB patients at different stages of liver inflammation and fibrosis and correlate these data with results from liver biopsies. Our purpose also included developing a model based on a combination of routinely measured clinical parameters along with CP levels to accurately predict fibrosis in patients with chronic hepatitis B-virus related liver disease.

## Materials and Methods

### Patients

This retrospective cohort study included a total of 198 treatment-naive patients chronically infected with HBV who were consecutively treated at the Liver Center, First Affiliated Hospital of Fujian Medical University between January 2009 and August 2012. All included patients were positive for hepatitis B surface antigen (HBsAg) for at least 6 months and all patients were positive for HBV DNA and had some level of fibrosis. Among exclusion criteria were: 1) presence of other types of viral hepatitis, hepatocellular carcinoma, alcoholic liver disease, decompensated cirrhosis, or autoimmune hepatitis; 2) concurrent infection with human immunodeficiency virus (HIV); 3) hereditary liver diseases; 4) drug-induced liver injury; 5) serum creatinine 1.5 times > ULN; 6) other factors that may influence CP levels such as nonalcoholic fatty liver disease, diabetes, and cardiovascular disease. Biochemical and virological data were obtained from patient serum samples. 

Our groups were split-sample with a concurrent validation group, in which the patients were randomly divided into two groups, one used to train/develop the rule and one used to validate it. That is, for purposes of this study, study subjects were randomly stratified into a training group (n=109) for the establishment of a predictive model of CP and its relationship to liver inflammation and fibrosis, and a validation group (n=89) for validating the predictive model. The study was approved by the Institutional Review Board of the First Affiliated Hospital, Fujian Medical University and written consent was given by the patients for their information to be stored in the hospital database and used for research.

### Liver histology and quantification of fibrosis

All liver biopsies were performed with the 16G Tru-Cut needle (TSK Laboratory, Tochigi-Ken, Japan) guided by color Doppler ultrasound (ACUSON, Aspen Advanced Ultrasound, Siemens Company, USA). Specimens of 15-20 mm liver tissues were fixed with 4% neutral formalin, paraffin-embedded and stained with hematoxylin-eosin-safran (HE) and Masson’s trichrome. A minimum of six portal tracts was required for diagnosis. Films were independently evaluated for diagnosis by more than one pathologist. Histological staging was based on the previously validated Chinese Guideline of the Programme of Prevention and Cure for Viral Hepatitis, 2000 [30]. This classification system uses 5 stages of liver inflammation (G) and fibrosis (S) to evaluate liver biopsy samples. The previously validated Chinese staging system is similar to the established algorithms of the Scheuer [31] and METAVIR [32] classification systems, which both apply the five stages for liver inflammation and fibrosis. All biopsy samples are classified as inflammation levels G 0-4 and fibrosis stages are classified as F0 (no fibrosis), F1 (mild fibrosis without septa), F2 (moderate fibrosis with few septa), F3 (severe fibrosis with numerous septa without cirrhosis) and F4 (cirrhosis) [32]. However, in the present study, we eliminated the F0 stage (patients without fibrosis were not included) and applied only F1, F2, F3 and F4.

### Serum parameters

CP was detected in venous blood using the nephelometric immunoassay kit (BN II System, Siemens Healthcare Diagnostics Products GmbH, Germany) with a lower limit of detection of 200 mg/L. Other serum biochemical parameters, which were determined within 1 week of liver biopsy, included total bilirubin (TB), alanine aminotransferase (ALT), aspartate aminotransferase (AST), gamma glutamyl transpeptidase (GGT), albumin, globulin, cholinesterase (CHE), total bile acid (TBA), α-fetoprotein (AFP), prothrombin time (PT), international normalized ratio (INR), white blood cell count (WBC) and platelet count (PLT).

All study patients were also evaluated for markers of HBV such as HBsAg, HBsAb, HBeAg, HBeAb, HBcAb (Abbott Co., Shanghai, China). Serum HBV DNA levels were determined at the First Affiliated Hospital, Fujian Medical University using a fluorescence quantitative PCR method (PG Co, Shenzhen, China). Patients with serum HBV DNA>500 IU/mL were referred to as HBV-DNA positive.

### Model development

After all measured biochemical markers were assessed for their ability to determine cirrhosis, comparative analysis of patients with and without cirrhosis was used to select appropriate variables for developing a predictive model for cirrhosis. After model development, the new model (APPCI) and four existing models (FI, APRI, GPI, and APGA) were analyzed by AUC for predictive value for cirrhosis.

### Statistical analysis

A total of 198 subjects were grouped into the training group and validation group at a ratio of 1.2 (55%:45%) through simple randomization using SPSS software. The demographics and characteristics of subjects in the two groups were summarized as mean±standard deviations (SD) for continuous data if the data followed normal distribution, and median with inter-quartiles (IQR: Q1 to Q3) if the data did not follow normal distribution; categorical data were summarized as n (%). Differences in continuous data between the training and validation groups were compared using the two-sample test if data followed normal distribution and Mann-Whitney U test if data did not follow normal distribution; differences in categorical data were compared using Pearson Chi-square test for gender, and Mann-Whitney U test for the ordinal inflammation stage and fibrosis stage.

Correlation analysis of inflammation stages and fibrosis stages with demographics and clinical characteristics of subjects in the training group was performed and represented using coefficient with corresponding *P*-value through Spearman’s correlation analysis or Kendall’s tau correlation analysis. The dispersion of CP value was summarized as mean±SD for the given subject’s demographics and characteristics. The association of CP value with the corresponding subject’s demographics and characteristics was performed using the two-sample t-test as well as the one-way ANOVA test, together with post-hoc pair-wise comparisons with Bonferroni adjustments. To identify the best cut-off of CP value for cirrhosis, ROC curve analysis was applied through univariate and multivariate binary logistic regression analysis. Some continuous variables without normally distribution were analyzed after logarithmic transformation for normality of distribution. Univariate logistic regression analysis was performed to analyze the odds ratio (OR) of significant factors associated with cirrhosis. Variables having a *P*-value <0.05 in the univariate analysis were selected and evaluated by multivariate logistic regression model using the conditional forward selection method. The best cut-off of CP value was selected based on the maximization of Youden index. The corresponding AUC, sensitivity, specificity, positive predictive value (PPV), and negative predictive value (NPV) were also represented along with the ROC curve analysis. Also, data of the validation group used to validate the model were chosen from significant data of the multivariate logistic regression analysis of the training group. All statistical assessments were two-tailed and *P*<0.05 was considered statistical significance. An adjusted significance level of 0.01 was considered for the multiple pair-wise comparisons. Statistical analyses were performed using SPSS 15.0 statistics software (SPSS Inc, Chicago, IL). Multiple ROC curves for five non-invasive models (APPCI, FI, APRI, GPI, and APGA models) were analyzed for predicting cirrhosis. Pair-wise comparisons of ROC curves were performed using MedCalc for Windows, version 9.38 (MedCalc Software, Mariakerke, Belgium).

## Results

Among 198 subjects (mean age 36.44 yrs; SD=10.59), there were 160 males (80.8%) and 38 females (19.2%). Patients’ demographic and clinical characteristics are presented in Table 1. All variables were consistent between the training and validation groups (all *P*-values>0.05). (Table 1).

**Table 1:** Demographic and clinical characteristics of subjects in the training and validation groups.

Variable	All	Training group	Validation Group	*P*-value
	(N=198)	(N=109)	(N=89)	
Age (yr)	36.44 ± 10.59	36.6 ± 10.75	36.25 ± 10.45	0.818
Gender				0.977
Male	160 (80.8)	88 (80.7)	72 (80. 9)	
Female	38 (19.2)	21 (19.3)	17 (19.1)	
Total bilirubin (mmol/L)	14.1 ( 10.18 , 20.28 )	14.2 ( 10 , 21 )	13.8 ( 10.2 , 18.65 )	0.55
Albumin (g/L)	41.62 ± 4.04	41.81 ± 4.03	41.38 ± 4.07	0.458
Globulin (g/L)	29.1 ± 4.37	29.31 ± 4.18	28.84 ± 4.59	0.451
ALT (IU/L)	66.5 ( 40 , 215.75 )	66 ( 40 , 200.5 )	69 ( 39.5 , 222.5 )	0.868
AST (IU/L)	50 ( 31 , 113 )	47 ( 32 , 120.5 )	51 ( 31 , 111.5 )	0.81
GGT(IU/L)	47.5 ( 25 , 86.25 )	48 ( 25 , 97 )	46 ( 25 , 78.5 )	0.367
TBA (mmol/L)	10.1 ( 5.08 , 22.7 )	11.7 ( 5.05 , 24.25 )	9.4 ( 5.15 , 18.1 )	0.7
CHE (IU/L)	7437.5 ( 6014.25 , 9372.5 )	7667 ( 5853.5 , 9485.5 )	7364 ( 6279.5 , 9239 )	0.653
PT (s)	12.7 ( 12.2 , 13.5 )	12.7 ( 12.2 , 13.5 )	12.7 ( 12.1 , 13.4 )	0.282
INR	1.04 ( 0.98 , 1.1 )	1.04 ( 0.99 , 1.11 )	1.03 ( 0.96 , 1.1 )	0.251
HBV DNA (logIU/ml)	5.63 ( 4.49 , 6.96 )	5.56 ( 4.47 , 7.2 )	5.66 ( 4.6 , 6.86 )	0.625
WBC (10^9^/L)	5.67 ( 4.78 , 6.57 )	5.62 ( 4.71 , 6.45 )	5.81 ( 4.88 , 6.83 )	0.501
PLT (10^11^/L)	189.39±53.23	185.09±52.40	194.66±54.06	0.209
AFP (ng/ml)	4.11 ( 2.55 , 12.89 )	4.45 ( 2.89 , 14.53 )	3.94 ( 2.43 , 8.85 )	0.186
CP (mg/L)	205.13±38.99	202.22±38.84	208.70±39.10	0.246
Inflammation stage				0.81
G1	35 (17.7%)	19 (17.4%)	16 (18.0%)	
G2	55 (27.8%)	28 (25.7%)	27 (30.3%)	
G3	79 (39.9%)	44 (40.4%)	35 (39.3%)	
G4	29 (14.6%)	18 (16.5%)	11 (12.4%)	
Stage of fibrosis, n (%)				0.15
F1	50(25.3)	26(23.8)	24(27.0)	
F2	58(29.3)	26(23.8)	32(36.0)	
F3	52(26.3)	32(29.4)	20(22.4)	
F4	38(19.2)	25(23.0)	13(14.6)	
Cirrhosis				0.139
No	160 (80.8)	84 (77.1)	76 (85.4)	
Yes	38(19.2)	25(23.0)	13(14.6)	

Continuous data were summarized as mean±SD if data followed normal distribution and as median (IQR: Q1 to Q3) if data did not follow normal distribution; ccategorical data were summarized as n (%).

Differences in continuous data between the training and validation groups were compared using two-sample test if data followed normal distribution and Mann-Whitney U test if data didn’t follow normal distribution; Differences in categorical data were compared using Pearson Chi-square test for gender, and Mann-Whitney U test for the ordinal inflammation stage and fibrosisstage.

TBA, Total bile acid; PT, Prothrombin time; WBC, White cell count; PLT, Platelet count.

^*^ *P*<0.05 indicates a significant difference between groups.

Table 2 shows the correlations between the inflammation stages and fibrosis stages and patients’ demographic and clinical characteristics in the training group (Table 2). Positive correlations were shown between inflammation stage and laboratory markers, including TB, globulin, ALT, AST, GGT, TBA, PT, INR, HBV DNA, and AFP. However, a negative correlation was shown between inflammation stage and markers, including albumin, CHE, WBC, PLT, and CP (all *P*-values<0.05). Positive correlations were also shown between fibrosis stage and markers, including TB, AST, GGT, TBA, PT, INR, and AFP. However, negative correlations were found between fibrosis stage and albumin, CHE, PLT, and CP (all *P*-values<0.05). Neither age nor gender showed significant correlation with either inflammation stage or fibrosis stage (Table 2).

**Table 2:** Correlation analysis of inflammation stages and fibrosis stages with subjects’ demographic and clinical characteristics.

	Inflammation stage			Fibrosis stage	
Variable	r	*P*-value		r	*P*-value
Age (yr)	-0.075	0.438		0.100	0.303
Gender	-0.049	0.578		-0.089	0.313
Male					
Female					
Total bilirubin (mmol/L)	0.264	0.006^*^		0.257	0.007^*^
Albumin (g/L)	-0.466	<.001^*^		-0.424	<.001^*^
Globulin (g/L)	0.271	0.004^*^		0.175	0.069
ALT (IU/L)	0.375	<.001^*^		0.118	0.223
AST (IU/L)	0.442	<.001^*^		0.194	0.043^*^
GGT(IU/L)	0.511	<.001^*^		0.348	<.001^*^
TBA (mmol/L)	0.439	<.001^*^		0.261	0.006^*^
CHE (IU/L)	-0.545	<.001^*^		-0.454	<.001^*^
PT (s)	0.547	<.001^*^		0.529	<.001^*^
INR	0.475	<.001^*^		0.451	<.001^*^
HBV DNA (Log_10_ IU/ml)	0.199	0.041^*^		-0.065	0.503
WBC (10^9^/L)	-0.041	0.038^*^		-0.062	0.523
PLT (10^11^/L)	-0.340	<.001^*^		-0.396	<.001^*^
AFP (ng/mL)	0.604	<.001^*^		0.494	<.001^*^
CP (mg/L)	-0.375	<.001^*^		-0.483	<.001^*^

Coefficients with respective p-values were derived through Spearman’s correlation analysis or Kendall’s tau correlation analysis.

* *P*<0.05 indicates a significant correlation.

Associations were shown between the mean CP value and gender, inflammation stage, fibrosis stage, and cirrhosis status for all subjects in the training group (all *P*-values<0.05). Males had lower CP values than females. Subjects with inflammation stage G4 had lower mean CP values compared to subjects with other stages. Subjects with cirrhosis had lower CP values compared to subjects without cirrhosis (*P*<0.001). CP values were significantly associated with inflammation stage, fibrosis stage, and cirrhosis status in males, but not in females (Table 3).

**Table 3:** Association of CP value with corresponding subjects’ demographic and clinical characteristics (N=109).

	Total(n=109)				Males(n=88)				Females(n=21)		
	n	CP value	*P*-value		n	CP value	*P*-value		n	CP value	*P*-value
Overall	109	202.22 ± 38.84	-		88	196.81 ± 37.98	-		21	224.90 ± 34.69	-
Gender			0.003^*^								
Males	88	196.81 ± 37.98									
Females	21	224.90 ± 34.69									
Age, yrs			0.378				0.920				0.523
<20	5	195.40 ± 43.49			5	195.40 ± 43.49			0	ND	
20≤age<30	24	203.29 ± 34.96			18	194.22 ± 26.93			6	213.86 ± 44.41	
30≤age<40	40	200.00 ± 35.80			33	197.06 ± 34.97			7	213.86 ± 39.21	
40≤age<50	28	196.82 ± 41.08			25	195.00 ± 42.43			3	212.00 ± 28.00	
age≥50	12	222.92 ± 47.98			7	209.71 ± 60.40			5	241.40 ± 11.26	
Inflammation stage			<.001^*^				0.001^*^				0.447
G1	19	211.37 ± 33.09			16	209.50 ± 34.44			3	221.33 ± 28.01	
G2	28	222.32 ± 43.41			20	214.95 ± 47.15			8	240.75 ± 26.29	
G3	44	196.59 ± 33.52			37	193.51 ± 30.15			7	212.86 ± 47.32	
G4	18	175.06 ± 31.13^abc^			15	167.20 ± 26.85^ab^			3	214.33 ± 20.31	
Fibrosis stage			<.001^*^				<.001^*^				0.526
F1	26	227.62 ± 32.26			17	222.24 ± 35.18			9	237.78 ± 24.45	
F2	26	208.73 ± 40.42			24	207.38 ± 40.70			2	225.00 ± 46.67	
F3	32	195.31 ± 33.63^d^			27	192.37 ± 31.37			5	211.20 ± 44.67	
F4	25	177.88 ± 33.43^d^			20	168.50 ± 24.91d^e^			5	215.40 ± 39.50	
Cirrhosis			<.001^*^				<.001^*^				0.497
No	84	209.46 ± 37.53			68	205.13 ± 37.25			16	227.88 ± 33.89	
Yes	25	177.88 ± 33.43			20	168.50 ± 24.91			5	215.40 ± 39.50	

CP values are represented as mean±SD for a given subject’s demographic and clinical characteristics.

Association was evaluated using two-sample t-test, or one-way ANOVA test with a post-hoc Bonferroni pair-wise comparisons.

* *P*<0.05, indicates a significant difference.

^abcde,^ indicates a significant difference compared to inflammation stage 1^a^, stage 2^b^, stage 3^c^, fibrosis stage 1^d^, stage 2^e^. (*P*<0.01)

ROC curve analysis was performed and the optimal cut-off CP value was determined for identifying stages of inflammation and liver fibrosis (Table 4). The AUC values were derived as 0.741 (95% CI=0.674 to 0.800) for inflammation stage G4 (G≥4), 0.754 (95% CI=0.688 to 0.812) for fibrosis stage F2 (F≥2), 0.712 (95% CI=0.644 to 0.774) for fibrosis stage F3 (F≥3), and 0.737 (95% CI=0.670 to 0.797) for fibrosis stage F4 (F≥4). According to the maximization of Youden index, the optimal cut-off values were 199 mg/L with sensitivity=82.76% and specificity=55.62% for G≥4; 204 mg/L with sensitivity=64.19% and specificity=80% for F≥2; 189 mg/L with sensitivity=55.56% and specificity=81.48% for F≥3; and 189 mg/L with sensitivity=65.79% and 71.87% for F≥4. (Table 4)

**Table 4:** CP values distinguish different stages of inflammation and fibrosis as measured by AUC (N=109).

Pathological stages	AUC (95% CI)	Youden index	Cut-off point	Sensitivity	Specificity
G≥4	0.741 (0.674, 0.800)	38.38	≤199	82.76%	55.62%
F≥2	0.754 (0.688, 0.812)	44.19	≤204	64.19%	80.00%
F≥3	0.712 (0.644, 0.774)	37.04	≤189	55.56%	81.48%
F≥4	0.737 (0.670, 0.797)	37.66	≤189	65.79%	71.87%

AUC, area under ROC curve; CI, confidence intervals.

### Model development

**Figure 1:**
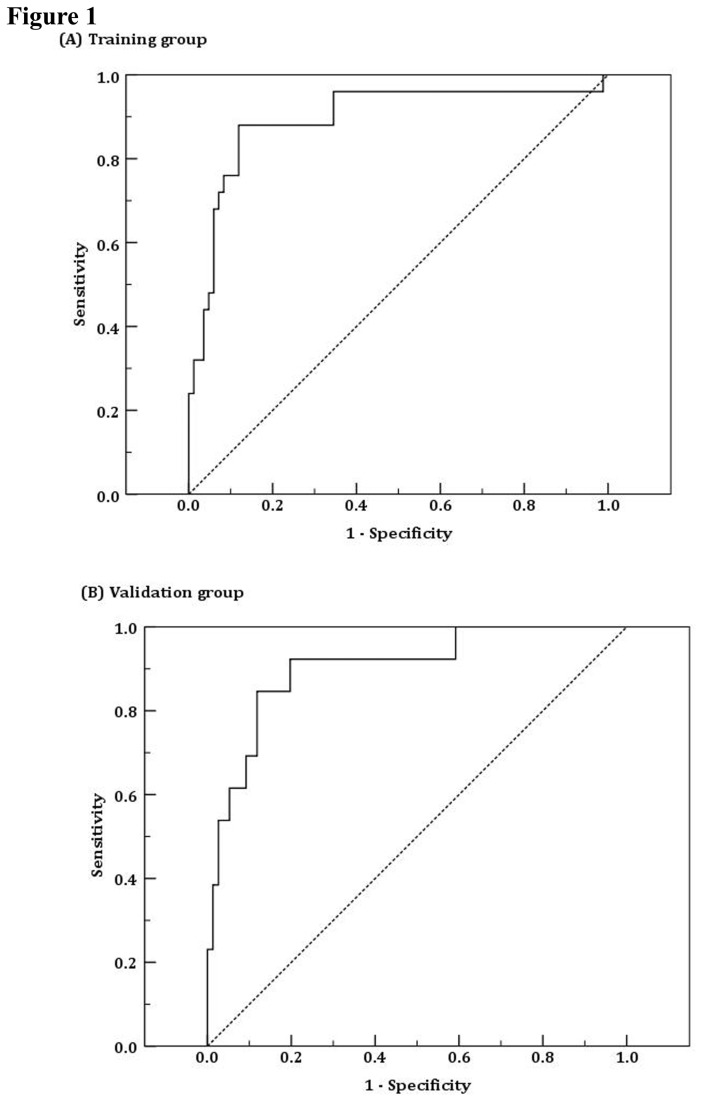
ROC curve of APPCI model in training (A) and validation (B) groups, respectively.

Univariate and multivariate logistic regression analyses were performed in order to identify factors associated with cirrhosis in the training group (Table 5). Univariate logistic regression analysis showed significant associations between cirrhosis and markers, including albumin, globulin, CHE, PT, INR, PLT, AFP, and CP (all *P*-values<0.05). Four markers (PT, PLT, AFP, and CP) were further selected for multivariate logistic regression analysis using the conditional forward selection method. The APPCI model was derived from the multivariate logistic regression analysis as: ⌜-28.89+1.157×LogAFP(ng/ml)+30.284×LogPT(s)-0.018×PLT(10^11^/L)-0.023×CP(mg/L)⌟, where APPCI was set as an APPC index from the APPCI model (this model is not presented in Table 5). The optimal cut-off for the predicted value from the APPCI model was selected as (-1.034) based on the maximization of the Youden index. The corresponding measurements were derived as AUC=0.893 (95% CI=0.820 to 0.944), sensitivity =88%, specificity =88.1%, PPV=68.7%, and NPV=96.1%. We also evaluated the APPCI model in the validation group. The optimal cut-off for the predicted value from the APPCI model was selected as (-1.127). The corresponding measurements were derived as AUC=0.904 (0.823, 0.956), sensitivity=84.62%, specificity=88.16%, PPV=55%, and NPV=97.10%, respectively (Table 6 and Figure 1).

**Table 5:** Univariate and multivariate logistic regression analysis to determine factors significantly associated with cirrhosis in training group (N=109).

	Univariate			Multivariate	
Variables	OR (95% CI)	*P*-value		OR (95% CI)	*P*-value
Age (yr)					
<20	Reference				
20≤age<30	0.800 (0.070 , 9.180)	0.858			
30≤age<40	1.517 (0.152 , 15.112)	0.722			
40≤age<50	1.091 (0.102 , 11.669)	0.943			
age≥50	1.333 (0.104 , 17.098)	0.825			
Gender					
Male	0.941 (0.307 , 2.888)	0.916			
Female	Reference				
Total bilirubin (Log_10_ mmol/L)	1.657 (0.348 , 7.899)	0.526		-	
Albumin (g/L)	0.792 (0.693 , 0.906)	0.001^*^		-	
Globulin (Log10 g/L)	1.171 (1.046 , 1.309)	0.006 ^*^		-	
ALT (Log_10_ IU/L)	0.892 (0.366 , 2.174)	0.802		-	
AST (Log_10_ IU/L)	1.133 (0.392 , 3.278)	0.817		-	
GGT(Log_10_ IU/L)	3.099 (0.915 , 10.500)	0.069		-	
TBA (Log_10_ mmol/L)	2.638 (0.958 , 7.269)	0.061		-	
CHE (Log_10_ IU/L)	0.010 (0 , 0.334)	0.010 ^*^		-	
PT (Log_10_ s)	1.3×10^12^ (2.7×10^5^ , 6.3×10^18^)	<.001^*^		1.4×10^13^ (4.4×10^4^ , 4.6×10^21^)	0.002^*^
INR (Log_10_)	4.5×10^9^ (9.5×10^3^ , 2.2×10^15^)	0.001		-	
HBV DNA (Log_10_ IU/ml)	1.006 (0.764 , 1.323)	0.969		-	
WBC (Log_10_ 10^9^/L)	0.034 (0.001 , 2.011)	0.104		-	
PLT (10^11^/L)	0.983 (0.972 , 0.994)	0.003 ^*^		0.982 (0.969 , 0.996)	0.011^*^
AFP (Log_10_ ng/ml)	3.940 (1.895 , 8.190)	<.001^*^		3.180 (1.321 , 7.656)	0.010^*^
CP (mg/L)	0.971 (0.955 , 0.988)	0.001^*^		0.977 (0.958 , 0.997)	0.022^*^

* significantly associated with cirrhosis. (*P*<0.05)

**Table 6:** Summary of validity of APPCI model in the training and validation groups.

	Training group	Validation Group
	(N=109)	(N=89)
AUC (95%CI)	0.893 (0.820 , 0.944)	0.904 (0.823 , 0.956)
Cut-off point	-1.034	-1.127
Accuracy rate		
Sen.	88%	84.62%
Spec.	88.10%	88.16%
PPV	68.70%	55.00%
NPV	96.10%	97.10%

Abbreviations: AUC, area under ROC curve; CI, confidence intervals; Sen., sensitivity; Spec., specificity; PPV, positive predictive value, and NPV, negative predictive value.

**Figure 2:**
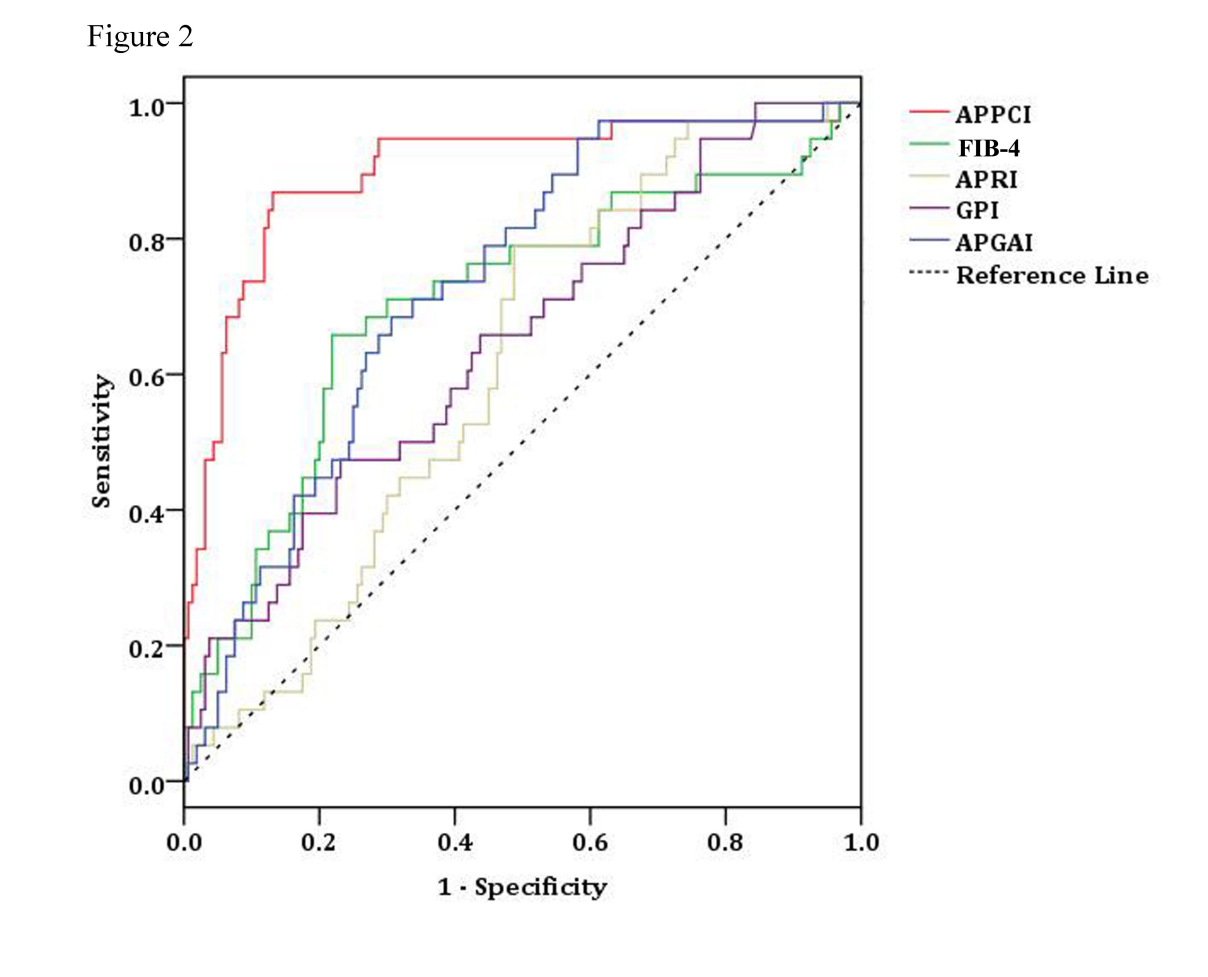
ROC curves of the APPCI, FI, APRI, GPI, and APGA noninvasive models in all study subjects.

ROC curves of the different non-invasive models were compared directly with that of APPCI in order to predict cirrhosis in the training and validation groups (Table 7, Figure 2). The APPCI model had a significantly greater AUC compared to the FIB-4, APRI, GPI and APGA models (all *P*-values<0.05; Table 7; Figure S1).

**Table 7:** Comparisons of different non-invasive models predictive of cirrhosis. (N=198)

Model^a^	AUC (95% CI)	Youden index	Cut-off point	Sen. (%)	Spec. (%)	PPV (%)	NPV(%)	*P*-value
APPCI	0.898 (0.847, 0.936)	73.71%	-1.1198	86.84%	86.87%	61.10%	96.50%	-
FIB-4	0.706 (0.637, 0.768)	43.91%	-31.6084	65.79%	78.12%	41.70%	90.60%	0.001^*^
APRI	0.610 (0.539, 0.679)	30.20%	2659.57	78.95%	51.25%	27.80%	91.10%	<.001^*^
GPI	0.645 (0.574, 0.711)	24.25%	156.23	47.37%	76.88%	32.73%	86.01%	<.001^*^
APGA	0.729 (0.661, 0.789)	37.79%	-4.1207	68.42%	69.37%	34.70%	90.20%	<.001^*^

^a^Models:

APPCI model: -28.89+1.157×(LogAFP)+30.284×Log(PT)-0.018×PLT-0.023×CP;

FIB-4 model: 8.0-0.01 × PLT (× 10^9^/L)-ALB (g/dL);

APRI model: AST (IU/L) × 100/PLT (× 10^9^/L);

GPI model: 1.7-0.01×100/PLT (× 10^9^/L)+0.5×Globulin (g/dL);

APGA model: 1.44+0.1490×log(GGT)+0.3308×log (AST)-0.5846×log(PLT)+0.1148log(AFP+1).

Abbreviations: AUC, area under ROC curve; Sen., sensitivity; Spec., specificity; PPV, positive predictive value, and NPV, negative predictive value.

*indicates a significant difference compared with APPCI model.

## Discussion

In this study, we investigated associations between serum CP levels and inflammation grade and liver fibrosis stages in patients with chronic hepatitis B-virus related liver disease. We showed that serum CP levels were negatively and indirectly correlated with inflammation and fibrosis stages in males. Additionally, we used a combination of routinely measured clinical parameters along with serum CP to establish a noninvasive model to predict and assess fibrosis and cirrhosis. 

CP, an alpha 2-glycoprotein that is mainly synthesized in the liver, has been shown to play a role in acute phase reactions in which serum CP levels are upregulated during inflammation and/or tissue damage [33]. Measurement of serum CP has been used to diagnose WD, a liver disease in which serum CP levels are decreased in up to 95% of symptomatic patients [34]. Although the levels of CP are downregulated in conditions like severe hepatitis, fulminant hepatitis and decompensated cirrhosis [29,35,36], the clinical significance of CP has not yet been clearly defined. In this study, we showed that there was no significant correlation between age and serum CP levels. However, a significant correlation was shown between CP levels and inflammation, and an increase in the number of necrotic hepatic cells seen with moderate or severe liver inflammation (G3-4) resulted in an obvious reduction of CP. We also showed that, although CP levels were normal at stages S1 and S2, levels declined significantly at stages F3 and F4. Patients with chronic liver disease typically experience repeated injury from severe necroinflammation and fibrogenesis, finally resulting in morphologically apparent fibrosis [4,8]. Our data suggest that moderate or severe inflammation results in lower levels of serum CP, since CP is synthesized in the liver. 

Although liver biopsy is currently the standard means of staging fibrosis in patients with CHB, the procedure is costly and invasive, carrying the potential risk of complications occurring during the procedure [11]. Biopsy results are also prone to variable interpretation of fibrosis, making it imperative to develop accurate and reliable noninvasive means to assess the severity of hepatic fibrosis and inflammation in HBV-related chronic liver disease. Among the noninvasive approaches used to assess liver fibrosis are the use of clinical symptoms and signs, routine laboratory tests (e.g., ALP, AST, ALT, GGT, PLT, PT); combined serum markers of fibrosis and inflammation, including the established models FIB-4, Fibrotest, APRI, ELF, Hepascore and Fibrometer, as mentioned and described above [15-19,21], as well as ultrasonography and radiological imaging studies [22,37-39]. Although these methods together are able to show various degrees of correlation with fibrosis stages, at present, none of these tests or markers alone is sufficiently accurate or reliable in predicting liver fibrosis. Liver stiffness measurement using transient elastography (TE) has also been applied as a measure of fibrosis, and has been shown to have high accuracy and good reproducibility while avoiding liver biopsy [40,41]. A recent meta-analysis of TE performance in 2772 cases of CHB had an AUC greater than 0.859 with high sensitivity and specificity, demonstrating that TE is suitable for assessing liver fibrosis in CHB patients [41]. The disadvantages of TE include that it requires a dedicated device, is unable to distinguish between intermediate stages of fibrosis, and its applicability is low in patients with obesity or ascites and when operators have limited experience [20]. In the present study, we used serum CP levels as a biomarker to identify CHB patients with fibrosis. Based on a scoring system for liver fibrosis established by the Chinese Medical Academic Association [30], which uses a 5-stage scoring system similar to the Scheuer [31] and MEDAVIR [32] classification systems, we showed that the AUCs for CP were 0.74 (G≥4), 0.75(F≥2), 0.71(F≥3) and 0.74 (F≥4). The ease of determining serum CP levels and its reliable correlation with liver inflammation and fibrosis in male patients suggests that using CP as a biomarker for fibrosis could potentially reduce the need for liver biopsies. Our findings give us a novel perspective by which to understand the sub-clinical meaning of CP. Although CP has been studied in conjunction with evaluating WD [27] and severe hepatitis [29], to the best of our knowledge, this is the first study evaluating correlations between serum CP and fibrosis in CHB, and the first study to use CP as a noninvasive predictor of hepatic fibrosis and risk of cirrhosis.

Based on the indirect association between the levels of CP and the stage of fibrosis in CHB patients, we used a combination of biochemical markers and serum CP levels to construct a model and a scoring system to distinguish patients with and without liver cirrhosis. Among the biochemical markers assessed, we showed that albumin, globulin, GGT, CHE, TBA, PT, INR, CP and PLT were predictors of cirrhosis. We used a comparative analysis of patients with and without cirrhosis and identified a combination of 4 variables (AFP, PT, PLT and CP) that we used in our predictive model, APPCI. Although AFP values are noted primarily for HCC diagnosis, they have been used previously as an indirect index to identify fibrosis stage [13,42,43]. PLT, the variable with the greatest AUC among the components of APPCI, correlates with the degree of portal hypertension and, to a lesser extent, with hepatic function and reduced thrombopoietin synthesis [44-46]. Similarly, PT, which is directly related to hepatic function, worsens with progression of fibrosis and loss of hepatocyte mass. The application of our formula to predict development of cirrhosis results in a simple score that can be used to select patients at low risk of having cirrhosis. Our APPCI model predicted liver cirrhosis with an AUC of 0.89, a sensitivity of 88% and specificity of 88.1%, a PPV of 68.7%, and NPV of 96.1% in the training group. In the validation group, the APPCI model predicted cirrhosis with an AUC of 0.90, a sensitivity of 84.6% and specificity of 88.1%, a PPV of 55.0%, and NPV of 97.0%. 

Existing noninvasive models, including FIB-4 index, FibroTest, ELF, APRI and Hepascore, were initially developed based on estimating fibrosis levels in patients with chronic hepatitis C, and therefore might not be suitable for CHB patients or may not have been validated in CHB [20]. Also, HCV and HBV infection may have different influences on the progression of fibrosis, differences in viral pathology and associations with related markers. APRI has been extensively studied in CHB and CHC patients and has been reported to be an easy, validated predictor of hepatic fibrosis in chronic hepatitis C [47]. FibroTest was the first algorithm that combined direct and indirect markers in a commercially available data scoring system, with an AUC of 0.87 in HCV patients [20]. In direct comparison with other laboratory markers of fibrosis, our predictive model, APPCI, had a significantly greater AUC (0.89) than FIB-4 (0.70), APRI (0.61), GPI (0.65) and APGA (0.73) models (all *P*-values<0.001). CP performed well as a single marker (AUCs 0.68 for F2 vs. F1, 0.76 for F3 vs. F1 and 0.86 for F4 vs. F1) (Table S1) and our new model compared favorably with previously developed models [48-52]. Park et al. [49] derived and validated a simple model of advanced fibrosis in patients with high viral load (HBV DNA) and elevated ALT, finding that age and AST were independent predictors of advanced fibrosis and sufficient to advise physicians about whether or not to perform liver biopsy. Kim et al. [51] evaluated various serum markers, finding that APRI was the most useful for predicting significant fibrosis, and that the cirrhosis discriminant score (CDS) was the most useful for predicting extensive fibrosis. Comparison of hyaluronic acid, galactose and methacetin breath tests, APRI, FibroTest and FIB-4 showed that all were able to distinguish between mild and moderate fibrosis and cirrhotic and non-cirrhotic patients, but not all were useful as single tests while combinations of tests increased accuracy [52]. The newly developed APPCI may have certain advantages, including no requirement for dedicated equipment or tests not offered routinely in most laboratories. Overall, combinations of tests still appear to have greater accuracy than single serum markers. 

Our study has a number of limitations. First, CP levels were measured at certain transverse sections of time, so the dynamic analysis of CP levels may possibly change the primary results obtained in this study. Second, since we recruited an unselected group of chronic HBV carriers, the number of patients with cirrhosis was relatively small (22.9%) and this could explain the relatively low PPV of our formula. In addition, we included far fewer female patients than male patients, thus it may explain why results for inflammation and fibrosis stages in females were not statistically significant; significant changes in fibrosis and inflammation were only in males and this finding must be investigated further in a larger gender-matched cohort. Third, although we performed internal validation using a randomly chosen cohort, our findings require prospective external validation. Also, our present work involves cross-sectional validation of a formula for predicting liver cirrhosis, however, longitudinal validation with serial liver biopsies would be required while using serum markers during follow-up of untreated patients or while evaluating the impact of treatment on liver injury [53]. Future study will include a larger gender-matched cohort, prospective design and further training of CP alone as a marker of liver fibrosis in CHB and of the related APPCI model, especially to determine why CP levels decline in CHB patients and to further validate the model. 

Despite the above limitations, our findings offer a promising noninvasive strategy that may circumvent the use of biopsies in patients who refuse or who have contraindications to liver biopsy. Results of this study present a new perspective on the expression differences of serological biomarkers for liver inflammation and fibrosis in HBV infection. The first part of this report was a retrospective cross-sectional study with correlation analysis of CP and liver inflammation and fibrosis. Results showed that CP correlated negatively with the degree of fibrosis in male CHB patients. The second part involved quasi-research to develop a non-invasive diagnosis of a fibrosis model for evaluating CHB patients and to conduct a preliminary validation. Rather than to prove the broader pathophysiological role of CP in serious liver disease, we hypothesized that CP can be applied effectively as a biomarker to help improve the non-invasive diagnosis of liver fibrosis in CHB patients and predict progression to cirrhosis. 

In conclusion, our data demonstrated a negative correlation between serum CP levels and liver inflammation and fibrosis stages in male CHB patients, suggesting that CP can be used as a biomarker to predict necroinflammation and fibrosis. Our model uses routinely assessed markers in combination with CP levels to reliably predict the probability of liver cirrhosis. The most important feature of our study was that we evaluated a consecutive series of treatment-naive CHB patients regardless of serum ALT and HBV DNA levels, and our formula included serum CP levels. Our model may therefore be helpful in reducing the number of biopsies required prior to treatment, and is an accurate and noninvasive way to assess the progression of liver cirrhosis.

## Supporting Information

Figure S1 **Individual ROC curves for all serum markers in the APPCI model.**
(TIF)

Table S1 **AUC values of APPCI and CP alone for determinations of F2, F3 and F4 versus F1.**
(DOC)
