# Supplementary figures and images for: Serum Ceruloplasmin Levels Correlate Negatively with Liver Fibrosis in Males with Chronic Hepatitis B: A New Noninvasive Model for Predicting Liver Fibrosis in HBV-Related Liver Disease
Source: PLoS One. 2013 Oct 25;8(10):e77942. doi: 10.1371/journal.pone.0077942 (PMC3837017; doi:10.1371/journal.pone.0077942)

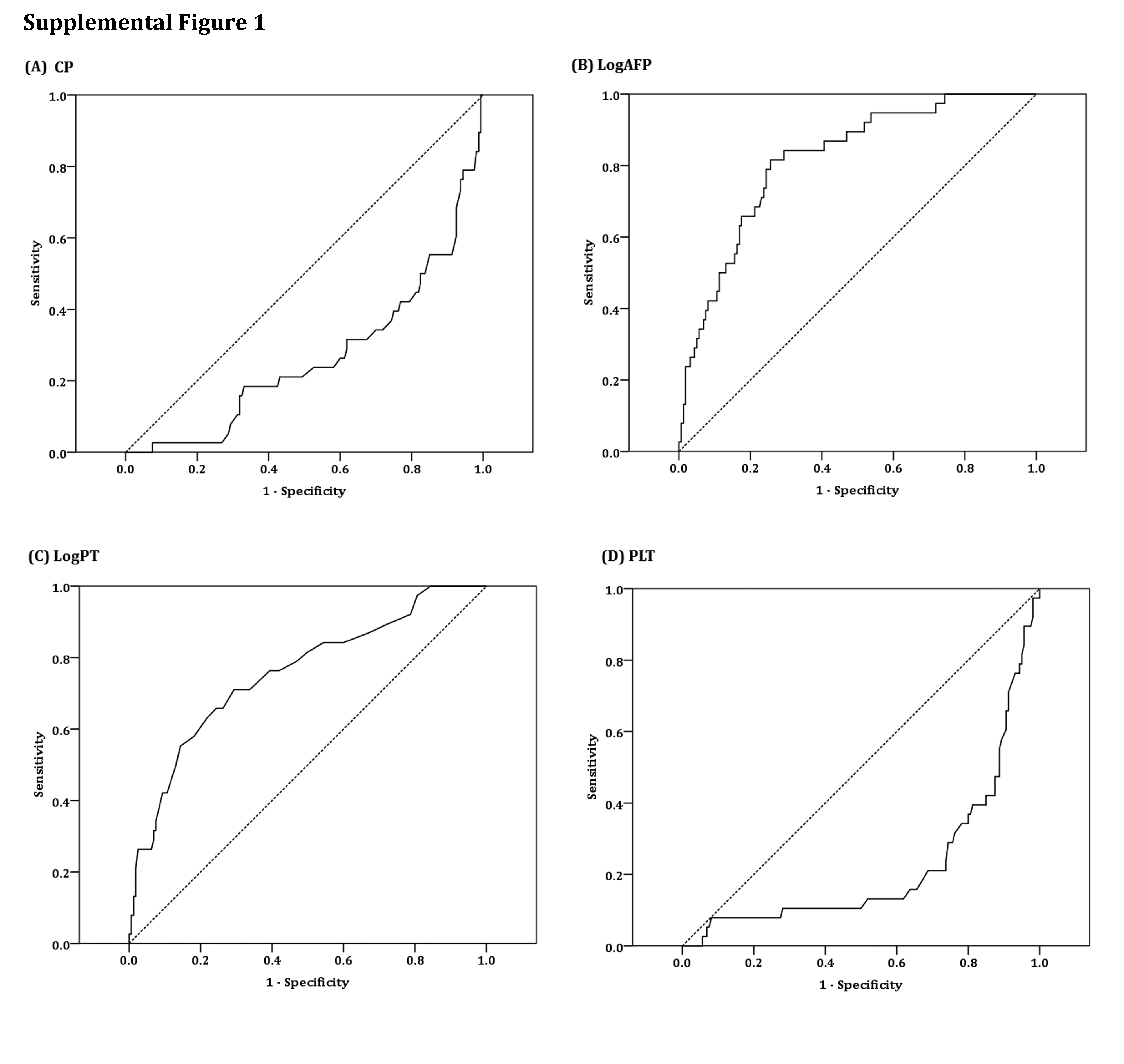

Supplement: Figure S1 — Individual ROC curves for all serum markers in the APPCI model. (TIF) [file pone.0077942.s001.tif]
