# Supplementary material for: Serum Ceruloplasmin Levels Correlate Negatively with Liver Fibrosis in Males with Chronic Hepatitis B: A New Noninvasive Model for Predicting Liver Fibrosis in HBV-Related Liver Disease
Source: PLoS One. 2013 Oct 25;8(10):e77942. doi: 10.1371/journal.pone.0077942 (PMC3837017; doi:10.1371/journal.pone.0077942)
Supplement: Table S1 — AUC values of APPCI and CP alone for determinations of F2, F3 and F4 versus F1. (DOC) [file pone.0077942.s002.doc]

Supplementary Table 1. AUROC values of APPCI and CP alone for determinations of F2, F3 and F4 versus F1

|  | F2 vs. F1 | F3 vs. F1 | F4 vs. F1 |
| --- | --- | --- | --- |
| APPCI model | 0.883 (0.795 – 0.971) | 0.931 (0.870 – 0.993) | 0.958 (0.884 – 1.000) |
| CP alone | 0.681 (0.532 – 0.830) | 0.760 (0.637 – 0.884) | 0.865 (0.764 – 0.966) |
